# Implications of self-care for health service provision

**DOI:** 10.2471/BLT.18.228890

**Published:** 2019-02-01

**Authors:** Manjulaa Narasimhan, Mukesh Kapila

**Affiliations:** a Department of Reproductive Health and Research, World Health Organization, avenue Appia 20, 1211 Geneva 27, Switzerland.; b Defeat NCD Partnership, United Nations Office for Project Services, Geneva, Switzerland.

Self-care interventions for health are among the most promising and exciting new approaches contributing to universal health coverage (UHC). The World Health Organization’s (WHO) working definition of self-care is “the ability of individuals, families and communities to promote health, prevent disease, maintain health, and to cope with illness and disability with or without the support of a health-care provider.”^1^ The scope of self-care in this definition includes health promotion; disease prevention and control; self-medication; providing care to dependent persons; seeking hospital/specialist care if necessary; and rehabilitation, including palliative care. This definition recognizes that individuals act to preserve health or respond to symptoms by determining their health-seeking behaviour and when to interface with professional care.^1^ Self-care is therefore comprised of actions within an individual’s control to manage health, including noncommunicable diseases or sexual and reproductive health.

With the increase of noncommunicable diseases such as diabetes, cancers, cardiovascular and chronic lung diseases, self-care can play a vital role in preventing and reducing underlying risk factors, optimizing treatment and managing complications.^2^ In this regard, the declaration of the Global Conference on Primary Health Care highlights the importance of empowering individuals as self-carers and caregivers.^3^ Self-care for noncommunicable diseases is not new and WHO has developed interventions for primary health care in low-resource settings.^4^ For instance, WHO recommends self-measurement to monitor blood pressure for the management of hypertension in patients where the affordability of the technology has been established.^5^ With increased access to drugs, diagnostics and devices converging with rapid advances in digital technologies, new configurations of self-care are made possible. Furthermore, as the power dynamics between doctor and patient shift to be more inclusive of the patients’ points of view, attention to human rights and health-sector accountability are essential to improve individuals’ autonomy for self-care.^6^ Health workers, including medical doctors, may be challenged by new forms of self-diagnostics and self-treatments for noncommunicable diseases and sexual and reproductive health. As health professionals integrate new technologies of self-care in the areas of testing, diagnostics, treatments and health maintenance into their practices, health care will change. Issues of shifting health costs, for health systems and individuals, and patterns of reimbursement will need to be addressed. On the other hand, self-care may be a preferred option in situations where patients are treated disrespectfully in health clinics, for instance women living with human immunodeficiency virus (HIV).^7^ Self-care interventions fulfil a particularly important role in these situations, as the alternative might be that people would not access health services at all. As individuals become increasingly able to take more active roles in their health management, people-centred approaches will become more acceptable and patients will be seen as active participants of their health and well-being.^8^

Improving access, equity and safety of health care lies at the heart of self-care. For instance, the potential of digital health in self-care is limited for vulnerable populations who may not be able to access the new technologies. Similarly, while pharmacies and pharmacists play an increasingly important role in providing information and services, factors such as out-of-pocket costs may not allow all those who need services and interventions to receive them. 

More research is needed to strengthen the evidence base. However, use of existing technologies could support various noncommunicable disease and sexual and reproductive health programmes, which could be delivered for instance through digital technology and mobile applications and be articulated in the context of self-care health.

The potential of self-care is that it can reach those who may not normally come to a health-care setting. Self-care can also support health goals in low-resource settings with fragile health-care systems. With increased cooperation between communities, health technology developers, practitioners and health ministries, multisectoral self-care interventions that meet people’s needs through comprehensive and integrated health services can be envisioned. As roles and responsibilities shift among different health practitioner groups, inter-professional communication will be vital.

WHO is developing a consolidated normative guideline around self-care, including for sexual and reproductive health and rights. Through a broad, consultative process, this guideline will build upon existing recommendations,^4^^,^^9^^,^^10^ including those for noncommunicable diseases, HIV self-testing and self-management of abortion. Self-care holds promise for reducing health inequities, potentially enhancing user autonomy and advancing UHC.
